# A survey with interventional components delivered on tablet devices versus usual care to increase pre-exposure prophylaxis uptake among cisgender Black women: a pilot randomized controlled trial

**DOI:** 10.1186/s12879-023-08019-z

**Published:** 2023-01-27

**Authors:** Mandy J. Hill, Angela M. Heads, Robert Suchting, Angela L. Stotts

**Affiliations:** 1 University of Texas Health Science Center at Houston, Houston, USA; 2 Department of Emergency Medicine, University of Texas Health Science Center at Houston, Houston, USA; 3 Department of Psychiatry and Behavioral Sciences, University of Texas Health Science Center at Houston, Houston, USA; 4 Department of Family Medicine, University of Texas Health Science Center at Houston, Houston, USA

**Keywords:** Pre-exposure prophylaxis, Sexual behavior, Cisgender Black women, Emergency department, HIV prevention, Linkage to preventive care

## Abstract

**Background:**

Cisgender (cis) Black women in the USA are more likely to become HIV positive during their lifetime than other women. We developed and implemented a behavioral intervention, Increasing PrEP (iPrEP), the first pilot randomized controlled trial (RCT) aimed at motivating cis Black women to be willing to use PrEP for HIV prevention and attend an initial PrEP clinic visit following an emergency department visit.

**Methods:**

Eligible participants were Black cisgender women ages 18–55 years who acknowledged recent condomless sex and substance use. Participants were randomized to iPrEP or usual care (UC). iPrEP is a survey-based intervention designed to raise awareness and knowledge about PrEP. Participants completed an assessment of knowledge of and willingness to use PrEP before and after the intervention, then received a warm-hand off with referral to a local PrEP clinic. Enrolled participants were followed for 6 months.

**Results:**

Forty enrolled participants were ages 18–54 years. Education levels varied evenly between some high school education and graduate education. Most participants were single (n = 25) or married (n = 7). Twenty-two participants were employed full-time. Pre-test results indicated that 21 of 40 participants had heard of PrEP. All participants identified PrEP as a daily HIV prevention medication. For those randomized to iPrEP, the odds of knowing about PrEP at post-test, when controlling for baseline, were higher relative to UC (OR = 5.22, 95%CrI = 0.50, 94.1]. iPrEP did not have any effect on willingness relative to UC. The estimate for iPrEP on willingness is marginally higher (4.16 vs. 4.04; i.e., 0.12 points higher); however, the posterior probability of 67.9% does not suggest a strong degree of evidence in favor of an effect. During the post-test, those receiving iPrEP were less ready to take PrEP than those receiving UC.

**Conclusions:**

Findings suggest that iPrEP increased knowledge about the PrEP medication but had a negative impact on readiness to take PrEP relative to UC. It is imperative that future research among cisgender Black women carefully considers the content provided in interventions designed to increase PrEP use, balancing the benefits of PrEP with the side effects and daily pill burden.

*Trial registration*: clinicaltrial.gov Identifier: NCT03930654, 29/04/2019.

## Introduction

Extending sexual health equity to populations with significant vulnerability to HIV requires a radical shift in access to preventive care for racial and ethnic minoritized populations. Specifically, cisgender Black women require the attention of capable and culturally sensitive practitioners who have significant awareness of relevant barriers to access and uptake of effective HIV prevention options like pre-exposure prophylaxis (PrEP). Actualizing equal access to PrEP requires interventions that can supplement resources to overcome structural barriers that prevent PrEP uptake to ultimately interrupt current patterns of HIV transmission.

Cisgender Black women are more likely to be newly diagnosed with HIV diagnoses than cisgender women in every other race/ethnic group [1, 2]. Black women comprise 13% of the United States (US) female population; yet, account for nearly 55% of all new HIV cases among US women [3]. Persistence in this trend demonstrates a pervasive health inequity that consistently aligns with an unmet prevention need for an often marginalized race and ethnic group in the US. The approval of Truvada as PrEP in 2012 presented an opportunity for equity in sexual health for sexually-active populations. However, a decade after PrEP was established as highly effective at preventing HIV transmission, it remains underutilized by an often-eligible population that could most benefit from uptake—cisgender Black women.

Structural and intersecting barriers to PrEP uptake among cis Black women include concerns of side effects, cost [4], poverty, structural racism, and unstable housing [5]. Sociocultural factors like stigma, medical mistrust, and avoidance by healthcare providers also serve as barriers to PrEP uptake among Black women [6, 7]. Hull et al. used vignettes about PrEP-eligible women with prescribing providers in HIV hotspot counties and tested willingness to discuss and prescribe PrEP with Black patients; findings revealed interaction between racial bias and patient race on providers’ perception of patients’ ability to adhere [8]. Bolstering utilization of PrEP by Black women requires interventions with capability to bridge the gap between providers and Black female patients so that the linkage to PrEP can happen.

In an effort to identify drivers of low uptake of PrEP among cisgender Black women, prevention scientists have evaluated perceptions, attitudes, readiness, and willingness of women to adopt PrEP as an HIV prevention strategy [9–14]. Willingness is a component of readiness to change and has been found to be predictive of future intentional behavior change [15]. A research study among women in Texas found that participants stated they were interested in PrEP and were willing to use it, but they were not current PrEP users [16]. This finding is similar to many formative research studies on PrEP willingness among cisgender Black women [17, 18]. However, PrEP willingness has not yet translated to a meaningful increase in PrEP uptake among cisgender Black women.

Brief, culturally-relevant and tailored interventions to improve motivation for actual PrEP uptake have been piloted [14]. The risk profiles of Black women vary amongst the studies. Dale [14] limited enrollment to Black women with an HIV positive sexual partner [14]. Variance in study designs (i.e. number of intervention and/or follow-up sessions) reflected differing degrees of involvement and commitment amongst study participants. Similarities in positive study outcomes were observed in relation to knowledge, motivation or willingness to take PrEP followed by assessing PrEP uptake, adherence, and barriers to uptake [14]. Although several prevention scientists have explored PrEP attitudes among Black women, many have yet to dedicate resources aimed at exploring barriers, overcoming barriers, and linking Black women to healthcare providers with the competencies needed to effectively link them to PrEP uptake and adherence. Consequently, opportunities to avert new HIV diagnoses continue to be missed. While this highlights an important gap in knowledge and sheds light on an inefficiency with public health practice, it also demonstrates the need to bridge PrEP access to HIV-vulnerable populations through innovative strategies that can connect with community members in a way that fosters the behavior change to include effective prevention practices. Investigators found that the sexual health prevention field requires attention to psychosocial and structural factors [14] that are specific to Black women in an effort to enhance motivation for PrEP and the health benefits of PrEP uptake in order to extend sexual health equity to cisgender Black women.

The authorship team lead a research study to bridge PrEP access to an HIV vulnerable population through the emergency department (ED). A systematic review by Gormley et al. concluded that nearly a third of ED patients who were enrolled as study participants were PrEP eligible [19]. Less than 50% of the PrEP-eligible participants indicated prior knowledge of PrEP. Although linkage to PrEP treatment or PrEP initiation happened, it occurred among a small sample of participants who expressed PrEP interest in the ED. Future research is necessary to identify strategies to increase PrEP education, interest, and linkage to care from the ED. Patients who seek healthcare in ED settings for non-emergent conditions are a hard to reach, primarily hidden population who typically do not have a primary care provider and therefore no point of entry for intervention. Given the disease burden of new HIV cases to cisgender Black women, intervening with this population in this setting has potential to fill a very important population health gap.

Through this paper, we present the study process and primary outcomes of the iPrEP study and the acceptability of the first use of a warm hand-off process aimed at linking cisgender Black women from the ED to local PrEP providing clinics. The researchers chose a survey-based intervention strategy because this strategy successfully increased willingness to PrEP in a one-arm pilot study and this intervention approach through a survey is more aligned with routine behavior and has a low likelihood of eliciting or reinforcing medical mistrust [20]. The hypothesis was that the iPrEP survey intervention would increase willingness to adopt PrEP among Black women recruited from an ED setting more than usual care (which does not include direct access to PrEP at the participating sites). If the hypothesis is proven, we would demonstrate evidence that the iPrEP intervention may be capable of bridging the gap in PrEP access for some PrEP-eligible cisgender Black women in the Southern US.

## Methods

**Fig. 1:**
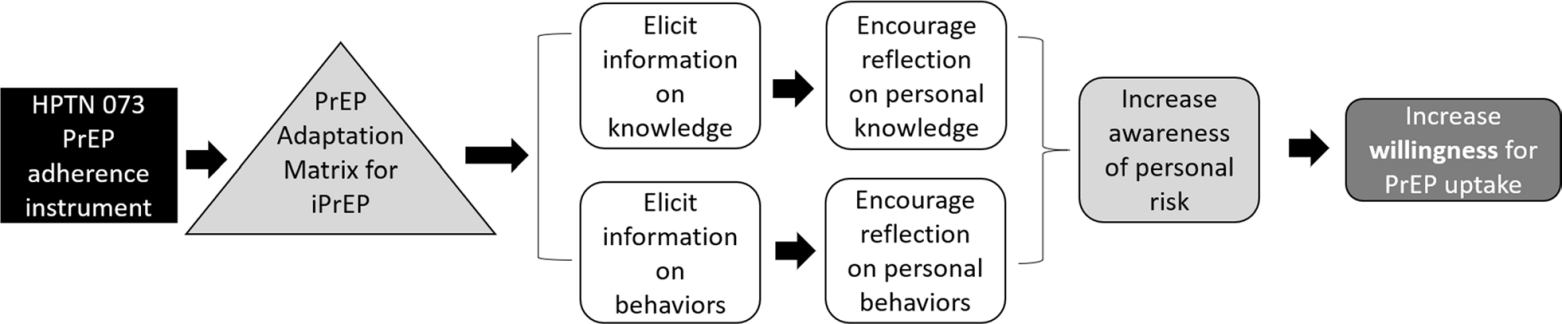
iPrEP impact intervention model

The study named ‘Leveraging the ED Visit to Increase Willingness for PrEP’ study (iPrEP study) is a parallel randomized controlled trial (RCT) with a 1:1 allocation ratio to an experimental or control group. The trial is registered at Clinicaltrials.gov (NCT03930654, 29/04/2019). The trial was conducted by research staff at the University of Texas Health Science Center at Houston (UTHealth), McGovern Medical School in partnership with two community clinics, Legacy Community Health and AIDS Foundation Houston. The institutional review board at UTHealth, The Center for Protection of Human Subjects, approved the study protocol for this trial (HSC-MS-16-0892). Study materials included variables in the screening REDCap database, informed consent, recruitment, intervention, and English-only electronic assessment forms developed using Qualtrics software. The experimental group mobilized the iPrEP intervention, a tablet-based intervention strategy that uses a survey model that aims to increase awareness of personal sexual risk through case scenarios (Fig. 1).

The control group consisted of usual care for mitigating behavioral health risks in the ED. Providing direct access to PrEP at the two sites is not a part of the usual care process. However, if a patient acknowledges substance use and is seen by the social worker, they have an opportunity to request a referral to PrEP services. The usual care by the social work team requires an in-person visit to each patient with active substance use during the ED visit. The social work team offers 24/7 services at both hospitals. These services include a needs assessments and referral for substance use treatment services. In many cases, several visits during the ED visit by the social work team are required, as patient responses direct referrals.

The study team led a 12-month RCT (August 2019–July 2021) to evaluate whether the iPrEP intervention coupled with a referral to a local PrEP clinic, relative to usual care, is capable of increasing willingness for PrEP uptake enough to prompt behavior change, specifically attendance to an initial PrEP clinic visit among cisgender Black women within a 6-month period, with a 1, 3 and 6-month follow-up [21]. There was a year-long lull from March 2020 to January 2021 in recruitment due to the COVID-19 pandemic. Recruitment procedures began again in March 2021.

The study established a new protocol of linking enrolled participants to a local PrEP clinic through a warm hand-off process co-managed by academic researchers and community-based PrEP providing agencies. The academic-community partnership was cultivated through mutual community-based work on a local PrEP advisory board led by the Houston health department. By linking enrolled participants to healthcare providers that are esteemed as pillars of the community, we enhanced access to social and contextual resources among cisgender Black women who were eligible for PrEP.

We recruited participants during wait times of an ED visit at two participating hospitals, Lyndon B. Johnson Hospital (LBJ) and Memorial Hermann Health System (MHH). Both hospitals are located in the Houston metropolitan area of Texas. LBJ is a public hospital and is the state’s busiest level 3 trauma center with more than 80,000 unique ED visits annually [22]. MHH is a private, not-for-profit, tertiary care center and has an annual ED census of approximately 75,000 patients per year. Study eligibility criteria (Table 1) were used to enroll Black women (N = 40) seeking care in the two participating EDs. We followed the standard criteria for confirming eligibility for using PrEP based on the Centers for Disease Control and Prevention (CDC) guidelines [23, 24]. Patient eligibility was confirmed through the electronic medical record (EMR) and trained researchers administered an in-person pre-screening survey using RedCap [25, 26] software on a tablet device. In addition to individuals who had an HIV test in the ED, individuals who were not offered an HIV test during an ED visit and stated that they received a negative result on a previous HIV test were also enrolled. Standard care for prescribing PrEP requires an HIV negative status.

**Table 1:** Eligibility criteria of cisgender Black women

Criteria	Description
Inclusion criteria	Cisgender women
	Race: Identified as Black or African American in the EMR
	Sexual orientation: Women who have sex with cisgender men
	Age: 18–55 years Does not decline an HIV test during the ED visit Current HIV negative status (based on the HIV test outcome in the ED and/or self-report)
	Acknowledges condomless sexual activity in the last three months Acknowledges substance use within the last three months
	Present to the ED with a non-emergent condition
	Basic understanding of how to answer survey questions on a tablet device
	Visual and comprehension capabilities PrEP eligible (based on established CDC criteria [23, 24]) Has a working mobile device with them Able to read and understand English sufficiently to provide informed consent and participate the study
Exclusion criteria	Currently taking medication with known contraindications for PrEP (brand name: Truvada) Currently on PrEP

When recruited patients were deemed eligible for study participation, a trained researcher engaged them in the informed consent process. The research team member explained the study, allowed the participants to read the consent form and ask any question, and obtained the participant’s signature. A copy of the signed consent form was given to the participant and a separate copy was added to the EMR. Enrolled participants were assigned a unique three-digit study identification (ID) number once the consent form was signed.

### Randomization process

Randomized assignments increase chances of obtaining groups that are comparable on salient baseline variables, even with a relatively small sample size. The randomize button, linked to a pre-loaded randomization schema prepared by the statistician, was selected after a study ID was assigned to each participant. While there is only one intervention session for participants randomized to the experimental arm, all other study procedures are uniform across both study arms over the 6-month period. Once randomized, study procedures began. Enrolled participants completed a pre-test on a tablet device.

### Description of study tools

#### Pre-test assessment

Directly after randomization took place, a pre-test instrument was loaded using the Qualtrics software [27] application on the tablet device. Each survey began with a text-entry of the study ID number, which allowed us to link survey responses to each enrolled participant. The pre-test assessed:

- Socio-demographics (age, education level, sexual orientation, income level, and employment status).
- Behaviors (sexual activity and substance use) – based on the risk assessment battery (RAB) [28–30].
- Predictive data (willingness to take PrEP to reduce risk of HIV).

### iPrEP intervention survey instrument

The experimental intervention, iPrEP, is based on an adaptation of the HIV Prevention Trials Network (HPTN) 073 study that was developed to assess structural and mental health factors that predicted PrEP uptake and adherence among Black men who have sex with men (MSM) [31]. The theoretical framework of the adaptation matrix used was rooted in the Theory of Gender and Power and the Sexual Script Theory in a concerted effort to create connection to the content and produce a culturally-competent and tailored intervention for cisgender Black women.

The team adapted the content and format of the HPTN 037 baseline instrument by integrating both an innovative intervention approach and a novel delivery platform using a tablet device. The intervention elements targeted increased awareness of sexual and substance use behaviors that place individuals at risk for acquiring HIV and promoted enrollment in PrEP programs among cisgender Black women seeking care in the ED [31]. iPrEP integrates brief, informational messages within a traditional survey to indirectly raise awareness of sexual risk behaviors and increase willingness for PrEP uptake. iPrEP increases knowledge through standard educational information about PrEP protocols, benefits, and side effects. This intervention aims to increase willingness for PrEP uptake among Black women seeking healthcare in an ED. iPrEP is grounded in behavioral willingness [32–34], a potentially stronger predictor of behavior than intentions or implicit attitudes [35]. Increasing willingness of cisgender Black women to take PrEP would theoretically increase the chances of follow-up to an initial PrEP clinic appointment after referral.

The original iPrEP instrument contained 88 unique questions. The format was updated, questions were collapsed and reformatted to include 41 questions, and the tool was adapted to improve ease of use. The iPrEP intervention uses qualitative themes and is divided into sections addressing factors with historical success at increasing PrEP adherence [36]. Specifically, the tool assesses personal concerns regarding HIV risks, quantity of male and female sex partners in the last three months, inquiries about relationship type (i.e. primary or main partner versus casual partners), length of relationships, awareness of partner’s HIV status, inquiries of recent transactional sexual encounters, types of sex with male and female partners, perceptions of people who are HIV positive, personal experience with HIV and STI testing, inquiries on substance use (i.e. quantity, frequency, type of substance), sexual encounters (i.e. condomless or with condoms) within two hours of substance use, perceptions on personal risk of contracting HIV, personal knowledge and awareness of PrEP, history with use of HIV prevention services, and personal confidence with condom use.

Participants’ thoughts on PrEP were evaluated using a 6-point Likert scale, with a ‘don’t know’ option, regarding PrEP’s ability to protect individuals from HIV with daily use, confidence with adherence to a daily pill regimen for HIV prevention, confidence in ability to schedule quarterly appointments with physicians, perception on PrEP reducing worry about condom use, willingness to take PrEP to prevent HIV and reduce the HIV burden on the African American community, willingness to take PrEP with small potential for side effects of nausea, vomiting, kidney problems, or loss of bone density. Scenarios to evaluate willingness to take PrEP using hypothetical scenarios were provided. Participant’s shared their current feelings about starting PrEP (i.e. readiness), and shared the reasons that they would use PrEP. Scales chosen to measure themes and sections are retained from the original HPTN 073 instrument. Scales are also modified (in some cases) for cultural competency and tailoring to cisgender Black women.

### Post-test assessment

Similarly to the pre-test, the post-test instrument begins with the study ID. The post-test is implemented after the iPrEP instrument/intervention is completed or a 5–10 min period after usual care takes place. The post-test assesses predictive data in two areas risk perception and willingness for PrEP uptake.

### Follow-up assessments

The follow-up assessment tool at each follow-up visit (1, 3, and 6 months) used the *Risk Assessment Battery* (RAB) score [28–30] and *TimeLine Follow-Back* (TLFB) [37, 38] to measure the frequency and persistence of risk during condomless sex and substance use. The RAB is a brief assessment of risk behaviors associated with HIV that is often used with substance-using populations. This assessment generally requires 15 min for completion. We also used the TLFB, the most reliable and valid method for assessing prior substance use [37, 39], to measure changes in substance use and sexual behaviors over the 6-month follow-up period. A revision of TLFB for assessing sexual activity and condom use was tested and refined in several CDC and NIH funded studies to reduce condomless sex and substance misuse (i.e. Project Choices) [37, 39] and was used in this pilot study. We used TLFB to measure changes in substance use and sexual behavioral practices between baseline and 1-month; 1-month and 3-months, and 3-months and 6-months. This tool was used in this pilot study to motivate participants to schedule and attend an initial or follow-up visit to the assigned local PrEP clinic [38, 40].

#### Data analysis plan

We established the feasibility of obtaining a sufficient sample of women from the ED [41–45] in prior studies; we achieved 100% of our planned accrual of cisgender Black women at both EDs. Thus, we estimated that recruitment of 40 cisgender Black women over a 12-month recruitment period was feasible.

Descriptive statistics were used to evaluate frequency, central tendency, and dispersion of sample characteristics. Generalized linear modeling (GLM) was used to evaluate follow-up measurements of each outcome (willingness to take PrEP, knowledge of PrEP, and readiness to take PrEP) as a function of treatment group, controlling for baseline. The primary outcome, willingness to take PrEP, was measured on a 5-point Likert-type scale from “strongly disagree” [1] to “strongly agree” [5]. The outcome was first modeled as a dichotomous variable with categories representing non-affirmative (“strongly disagree,” “disagree,” or “neutral”) and affirmative (“agree” or “strongly agree”) responses via the Bernoulli distribution (akin to logistic regression), followed by modeling the full range of response options via the Gaussian distribution. Knowledge of PrEP (“yes” vs. “no”) was modeled via the Bernoulii distribution (akin to logistic regression) and readiness to take PrEP was modeled via the Gaussian (normal) distribution. Sample characteristics (e.g., age; education; employment status) were screened as potential confounders via guidelines in the literature [46, 47]. Any characteristic demonstrating a relationship with both treatment and a given outcome would be included in the final model for that outcome as a covariate, if inclusion resulted in different inferences from exclusion. No sample characteristics met criteria for confounding in the present analyses.

Bayesian statistical inference was used to quantify the chance that an effect of treatment group exists (i.e., the alternative hypothesis) on each outcome, adjusted for baseline. Interactions between treatment and baseline was assessed for knowledge, willingness, and readiness. The Bayesian approach was pre-specified in the protocol to provide an accessible account of the probability of a treatment group difference even in the context of a smaller sample size [48]. For all models, weakly informative priors (b ~ N(mean = 0, sd = 10)) were used to provide robust, regularized estimates of model parameters and maximize the influence of the observed data on posterior probabilities. Assumptions of Bayesian analyses (i.e., convergence measure *R-*hat < 1.01 for all model parameters; sufficient effective sample size; posterior predictive checking to support model fit) were checked and determined to be satisfied for all analyses.

Given the observed data and weakly informative priors, Bayesian analyses provide a range of probable estimates called the *posterior distribution* for each model parameter. The median of the posterior distribution was taken as a most likely point estimate of the effect, and the highest probability range of values was taken as the 95% of the posterior distribution around that point estimate (i.e., the 95% credible interval, or CrI). The posterior distribution also provided the posterior probability (PP) than an effect of the intervention exists, expressed as the range of values in the posterior distribution that were greater or less than zero for the regression coefficient of treatment (i.e., PP (b > 0) or PP(b < 0)). For exponentiated coefficients (e.g., odds ratios (OR)), the existence of an effect was expressed relative to OR = 1 (i.e., PP (OR > 1) or PP (OR < 1)).

Decision making in the Bayesian context requires researchers to make judgments based on their subjective interpretation of a meaningful threshold. Heuristics from the literature [49–51] have provided probability thresholds that denote different degrees of evidence: none (PP = 50%), anecdotal (51–74%), moderate (75–90%), strong (91–96%), very strong (97–99%), and extreme (> 99%). For the current study, a pre-specified threshold was stipulated whereby an intervention effect that demonstrated at least 75% probability of increasing the odds of willingness to attend a PrEP clinic visit by a factor of 1.25 (i.e., PP(OR ≥ 1.25) ≥ 75%) would merit further investigation in a larger trial.

## Results

### Demographics

The current sample included N = 40 women (20/group) of mean age M = 33.7 (sd = 8.8). Detailed sample characteristics between and within treatment groups are provided in Table 2. Group differences with respect to the continuous variable (age) were evaluated via logistic regression, and differences with respect to the categorical variables were evaluated via Fisher’s exact test.

**Table 2:** Demographic description of the study population (n = 40 Black women)

Characteristic	Overall	iPrep	Usual care	*p*-value
Age	33.67 (8.83)	32.88 (7.26)	34.37 (10.18)	0.610
Education				0.621
Some high school	8 (21%)	4 (21%)	4 (20%)	
High school or GED	13 (33%)	7 (37%)	6 (30%)	
Some college	10 (26%)	3 (16%)	7 (35%)	
College graduate	1 (2.6%)	1 (5.3%)	0 (0%)	
Graduate education	7 (18%)	4 (21%)	3 (15%)	
Sexual orientation				> 0.999
Heterosexual	29 (74%)	14 (74%)	15 (75%)	
Homosexual	1 (2.6%)	1 (5.3%)	0 (0%)	
Bisexual	9 (23%)	4 (21%)	5 (25%)	
Marital status				0.081
Married	7 (18%)	2 (11%)	5 (25%)	
Separated	1 (2.6%)	1 (5.3%)	0 (0%)	
Divorced	6 (15%)	1 (5.3%)	5 (25%)	
Never married	25 (64%)	15 (79%)	10 (50%)	
Income				> 0.999
< $500	7 (18%)	3 (17%)	4 (20%)	
$501–$1000	12 (32%)	6 (33%)	6 (30%)	
$1000–$1500	5 (13%)	3 (17%)	2 (10%)	
$1500–$2000	3 (7.9%)	1 (5.6%)	2 (10%)	
> $2000	11 (29%)	5 (28%)	6 (30%)	
Employment status				0.751
Employed	22 (56%)	10 (53%)	12 (60%)	
Unemployed	17 (44%)	9 (47%)	8 (40%)	

### Retention and recruitment outcomes

**Fig. 2:**
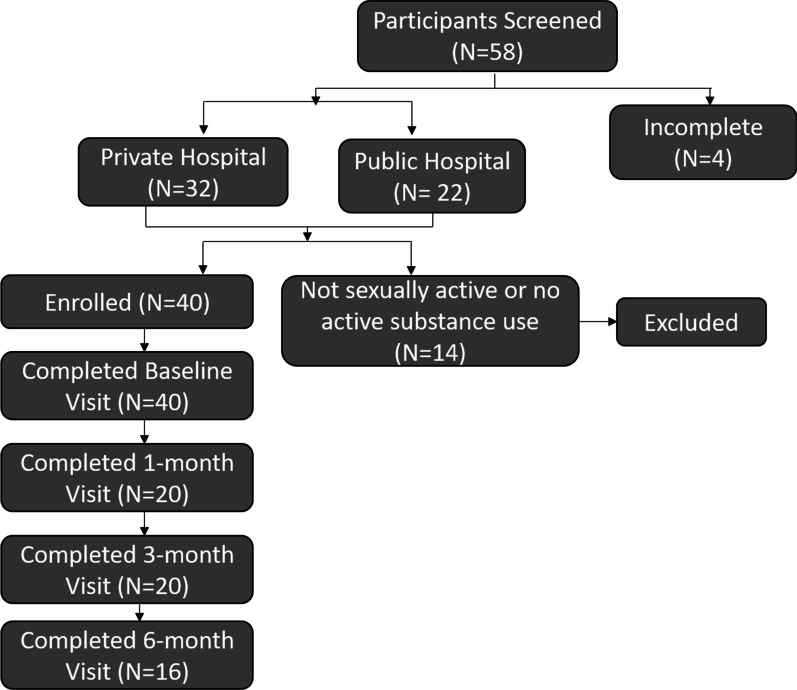
Participant recruitment and retention outcomes

Of 40 participants enrolled, retention rates at each follow-up visit were as follows: 50% at 1-month, 50% at 3-months, and 40% at 6-months (Fig. 2). A minority (27.5%, n = 11) of participants completed all three follow-up assessments.

### Knowledge of PrEP

Knowledge of PrEP at follow-up was also modeled as a function of treatment group and baseline in Table 3. Analyses supported an overall treatment effect (OR = 5.22, 95% CrI [0.50, 94.1]; PP(OR > 1) = 90.6%), adjusted for baseline. Although the wide credible interval reflected a lack of precision in estimation, the majority of the posterior distribution supported the existence of the effect. A follow-up model examined knowledge as a function of treatment, baseline knowledge, and the interaction between treatment and baseline knowledge. This model supported the existence of the interaction (PP(OR < 1) = 89.4%), suggesting that the effect of treatment was moderated by baseline knowledge. The probability of having PrEP knowledge at follow-up was relatively high for all individuals who reported having PrEP knowledge at baseline (usual care: 87%; iPrEP: 94%). Conversely, the probability of having PrEP knowledge at follow-up was higher (PP = 96.6%) across treatment groups for those who reported no PrEP knowledge at baseline (usual care: 50%; iPrEP: 99%).

**Table 3:** Description of reported predictors of PrEP use

Pre-test						Post-test			
Study arm		iPrEP		Usual care		iPrEP		Usual care	
Categories	Subcategories	N = 20	%	N = 20	%	N = 20	%	N = 20	%
PrEP knowledge									
Have you ever heard of PrEP?									
	Yes	14	70.0	7	35.0	14	70.0	12	60
	No	4	20.0	13	65.0	2	10.0	7	35.0
	Missing	2	10.0	0	0.0	4	20.0	1	5.0
PrEP willingness									
I would be willing to take PrEP to reduce my own risk of getting HIV									
	Strongly agree	7	35.0	5	25.0	7	35.0	6	30.0
	Agree	3	15.0	6	30.0	5	25.0	8	40.0
	Neutral	5	25.0	5	25.0	3	15.0	3	15.0
	Disagree	1	5.0	2	10.0	1	5.0	1	5.0
	Strongly disagree	3	15.0	2	10.0	1	5.0	1	5.0
	Missing	1	5.0	0	0	4	20.0	1	5.0
PrEP readiness									
In thinking about PrEP, an HIV medication taken BEFORE having sex, which of the following best describes your current feelings about starting PrEP?									
	1. I am not thinking about starting PrEP at this time	6	30.0	5	25.0	4	20.0	4	20.0
	2. Prevention by using PrEP is important to me, but I am not ready to start it yet	3	15.0	5	25.0	5	25.0	6	30.0
	3. I have thought about starting PrEP, but I have not yet tried to find a doctor or clinic	9	45.0	6	30.0	6	30.0	5	25.0
	4. I have found a doctor or clinic that provides PrEP but have not yet tried to make an appointment	0	0	3	15.0	2	10.0	1	5.0
	5. I have tried to obtain PrEP from a doctor or clinic but have not yet been successful	0	0	0	0	0	0	0	0
	6. I have an appointment for PrEP with a doctor or clinic but have not yet been there	1	5.0	1	5.0	0	0	3	15.0
	7. I have already gone to a doctor or clinic to obtain PrEP once	0	0	0	0	0	0	0	0
	Missing	1	5.0	0	0	3	15.0	1	5.0

### Willingness to take PrEP

Willingness to take PrEP at follow-up was modeled as a function of treatment group and baseline in Table 3. First, the outcome was modeled as a dichotomous variable with categories of non-affirmative and affirmative: analyses did not find support for an effect of treatment at the a priori effect size and PP threshold (PP (OR > 1.25) = 51.5%; i.e., lower than the 75% threshold). An exploratory follow-up model then evaluated the outcome across the full range of response options and a test of the null effect; this model also did not support an effect of treatment on willingness to take PrEP (*b* = 1.28 [− 5.70, 8.84]; PP (b > 0) = 69.9%). Further analyses did not find evidence supporting a moderating effect of baseline willingness on the relationship between treatment and follow-up willingness (*b* = − 0.36 [− 11.11, 11.30]); PP (b < 0) = 52.8%).

### Readiness to take PrEP

Readiness to take PrEP at follow-up was modeled as a function of treatment group and baseline in Table 2. Analyses found moderate support for a treatment group difference with respect to readiness at follow-up (PP(b < 0) = 85.4%) such that participants in the iPrEP condition demonstrated lower readiness at follow-up than participants receiving usual care (b = − 1.05, 95% CrI [− 5.63, 1.42]). A follow-up model examined readiness at follow-up as a function of treatment, baseline readiness, and the interaction between treatment and baseline readiness. This model supported the interaction between baseline readiness and treatment with moderate evidence (PP(b < 0) = 79.3%). Baseline readiness was positively related to follow-up readiness across groups; however, the average trend was stronger for individuals receiving usual care (b = 3.89 [1.39, 12.80]) relative to those receiving iPrEP (b = 2.58 [0.73, 10.57]).

### Warm-hand off to local PrEP clinics

Most enrolled participants (97.5%; 39/40) engaged in the warm hand-off process following the RCT and received a referral to one of two local PrEP clinics. One participant withdrew from the study prior to the warm hand-off process. A total of 38 referrals were made. One participant declined the referral to the PrEP clinic and another participant was being hospitalized and perceived that she could not make the appointment; however, the researcher provided the participant with the information needed to make the appointment upon discharge. A total of 37 clinic appointments were made during the ED visit. Only two participants (5.4%, 2/37) who received a PrEP clinic appointment actually self-reported a linkage to an initial PrEP clinic visit. Participants were more likely to choose AIDS Foundation Houston as their PrEP clinic of choice compared to Legacy Community Health, 64.86% (24/37) versus 35.14% (13/37), respectively. Few (7.5%, n = 3) participants had a sustained willingness to attend an initial PrEP clinic visit and schedule an additional clinic appointment during the follow-up period. We received confirmation, regarding attendance to an initial PrEP clinic visit, from our partners at two local PrEP clinics, for only one participant. Of the study participants who did not follow-up to the PrEP clinic appointment, the reasons included technical errors, clinic scheduling, desire for care elsewhere (i.e. had an established gynecologist or clinical home for women’s health), suboptimal experience with ED staff, relocation, other obligations, other medical conditions, and failure to remember the appointment. Two participants stated that they were willing to make a new PrEP clinic appointment during the follow-up visits.

#### Attendance to local PrEP clinic and PrEP uptake

Although two participants self-reported an initial PrEP clinic visit, only one was verified with our linking data point, which was the referral code. The linked patient received a PrEP prescription and reported PrEP uptake and adherence at the 6-month follow-up visit.

## Discussion

Engaging vulnerable populations, who are seeking primary care services in the ED, into prevention services to preserve their sexual and reproductive health is necessary. Mobilizing innovative approaches to extend PrEP access and promote PrEP uptake is an engagement approach with potential to actualize a meaningful decrease in new HIV cases at the population level. To our knowledge, and based on an extensive literature review, this is the first pilot study in the US to initiate a RCT aimed at increasing willingness for PrEP followed by an actionable step, a real-time linkage to a PrEP clinic from the ED visit. This is also the first RCT to move beyond assessing knowledge and attitudes about PrEP among cisgender Black women. The research process here builds self-efficacy for cisgender Black women to access PrEP within a 72-h period by scheduling an appointment in real-time, during an ED visit, with culturally-competent healthcare providers who are ready to initiate PrEP access and uptake. Study findings presented here make a significant contribution to the growing body of literature aimed at increasing PrEP uptake among HIV-vulnerable populations.

The iPrEP intervention was superior to usual care at increasing knowledge about PrEP to enrolled participants. Essentially, those who did not know about PrEP before the study were much more likely to know about it after the study if they received the iPrEP intervention, rather than usual care. Previous qualitative research approaches found a correlation between informing cisgender women about PrEP and positive increases in PrEP attitudes and interest [52]. Researchers predict that translating this correlation through interventions that reflect women’s preferences and perceived barriers to PrEP is a critical step towards increasing PrEP uptake among cisgender women [52, 53]. Similar qualitative approaches using focus groups discerned low PrEP awareness among cisgender women whereby participants perceived the importance of PrEP as a way to foster PrEP uptake and adherence [53]. The iPrEP intervention tested whether the correlation between increasing PrEP knowledge would correlate with enhanced attitudes toward and interest in PrEP, which we hypothesized would then translate to PrEP willingness, readiness, and uptake. The iPrEP intervention did indeed increase knowledge among participants more than usual care and the knowledge increase sustained across the follow-up period. However, the increase in knowledge did not correlate with a meaningful change in PrEP willingness and PrEP readiness decreased after exposure to iPrEP. In summation, a heightened awareness of the side effects to PrEP likely fueled fears about PrEP [54–56] The fear may have overshadowed perceptions on the potential health benefit of PrEP uptake and adherence.

As the iPrEP intervention, with 99-unique questions, contributed to an increase in willingness to use PrEP among 69% of 15 cisgender Black women during a single-arm development study [16]; we hypothesized that the increase in willingness would persist with a refined 41-item iPrEP intervention when implemented in an RCT compared to usual care. However, this was not the case. Despite increases in knowledge, the intervention had no effect on willingness to use PrEP. Failure of a revised and refined iPrEP intervention to duplicate effectiveness at increasing willingness for PrEP when compared to usual care is surprising and is likely correlated to the hyperfocus of the knowledge content on the side effects of PrEP instead of a balanced focus on both benefits and risk of PrEP as an HIV prevention intervention. Also, willingness in the previous single arm study of iPrEP was not acted upon. All participants in the RCT were required to pre-emptively agree to receiving a referral to the PrEP clinic during the pre-screening period. The perceived differences in taking PrEP ‘some-day’ versus ‘taking PrEP, possibly within a three-day period’, may have resulted in different answers. Reluctance may have become more pronounced after learning detailed information regarding side effects of the prevention regimen in the iPrEP study arm.

Apart from findings on willingness, we discerned an intervention effect regarding knowledge. Participants had higher knowledge at follow-up when compared to baseline. Importantly, knowledge was higher among participants randomized to iPrEP (94%) compared to those randomized to usual care (87%). However, the chance of demonstrating PrEP knowledge at follow-up was different for individuals who reported no PrEP knowledge at baseline. Chandler et al. developed a pilot PrEP education intervention and enrolled 43 Black college women. Their study findings illustrated that 67% of the cohort had not heard about PrEP and some (72%) were apprehensive to initiate PrEP; however, 67% of enrolled participants found the intervention useful and self-reported an increase in knowledge [57]. Hirschhorn et al. surveyed 370 HIV negative cisgender women, 83.0% were Black/African American, and only 30.3% had heard of PrEP. After learning about PrEP, 25% of participants stated they would consider starting PrEP [52]. These two recent studies suggest that recent interventions are increasing the knowledge of cisgender Black women regarding PrEP, and have potential to increase considerations for PrEP. Yet, neither of these studies measured how an increase in knowledge would translate to PrEP readiness or PrEP uptake. Differences in the iPrEP intervention’s effect on knowledge versus willingness shows that intervention has educational potential, but has room for improvement with its ability to motivate actionable metrics of behavior change, including PrEP readiness and PrEP uptake.

Participants in the usual care group reported a higher level of readiness for PrEP uptake than individuals who received the iPrEP intervention. Participants who received the iPrEP intervention learned more about the nuances of PrEP uptake, with specificity to side effects that can impact liver function, cause nausea, and possibly have a negative effect on bone density. Individuals in the usual care arm did not receive this information at all. Thus, each group made decisions about readiness based on different information. Hirschhorn et al. stated that 81.1% of their participants had concerns about taking PrEP with side effects as a common concern [52].

We implemented a new and actionable step that afforded the opportunity to translate PrEP knowledge, willingness, and readiness into an action, an initial PrEP clinic visit. The warm hand off process was based on the standard of care referral process for clinical care. Individuals who receive care in the ED are routinely referred to other clinical care settings for follow-up care [58]; however, this process had not been used for preventive care, until now. We established an innovative stepwise process to link PrEP-eligible cisgender Black women to one of two partnering local PrEP clinics [59, 60]. Most (39/40) enrolled participants were offered an opportunity to make an informed decision on which PrEP clinic they wanted to attend for their initial PrEP clinic appointment based on geography and specialized services (i.e. same day PrEP, women’s health services, weekend hours, etc.) offered by each clinic. Designated clinic personnel engaged us in conversation during the ED visit and often scheduled the PrEP clinic visit in real-time. Based on reported reasons for failure to attend the initial PrEP clinic visit among 94.6% of appointments made, social and structural challenges were stronger barriers than the facilitators we integrated within our study protocol (i.e. transportation, gift cards, warm hand-off process through partnerships with local PrEP clinics).

Although the findings of the pilot RCT did not prove the null hypothesis, the warm hand-off process was deemed feasible and largely acceptable by the participant population. Hill and Coker affirmed that the ED is an ideal clinical environment for brief interventions that promote sexual health among cisgender Black women presenting with nonemergent health conditions [61]. Providers have effectively linked ED patients to primary prevention strategies that promote clinical health [44, 45, 62–70]; however, it is time to extend that work to link ED patients to sexual health prevention services, potentially as a usual care practice.

### Limitations

The generalizability of study findings are limited in the following ways. There was a significant change in the staff, requiring re-training and interruptions with enrollment. We paused enrollment for 10 months during the COVID-19 pandemic in order to minimize risk to the research staff and the patient population. Injectable PrEP (i.e. cabotegravir) was not yet approved during the enrollment period of the study. It is likely that presentation of PrEP as a daily pill option alongside a bi-monthly injection option may have had a positive influence on readiness and willingness in the intervention. Both options may have also had a positive influence on clinic attendance and PrEP uptake to varying degrees across study arms.

During the warm hand-off process, the phone signal in the hospital sometimes prevented real time connections between research staff and hospital partners. This could hinder motivation and engagement to attend the initial PrEP clinic visit. We utilized alternative strategies to make the connection between the PrEP clinic and the patient (i.e. changing location within the hospital) in order to foster a real-time connection, but this approach was met with logistical challenges. Our methodology of relying on the participant to provide the referral code to the PrEP clinic during the initial clinical visit became a significant barrier to confirming a secure link, as the protocol did not allow interagency sharing of protected health information. The referral code could not be verified by the partnering agency in at least one case. It is likely that some of our participants attended initial PrEP clinic visits, but were not accounted for because they were received by clinic staff as patients instead of research participants.

### Future research

Efforts to bridge connections between PrEP-eligible women at significant risk for HIV and healthcare providers of those women should involve a person-centered approach with tailoring to providers and patients with educational and behavioral intervention elements that can be delivered with cultural competency and sensitivity with a goal of strengthening links between primary care settings and ED settings to comprehensive sexual health services with local partners [71–74]. More research is needed to provide evidence that clinical settings offering care to PrEP-eligible cisgender women can integrate PrEP access into current care protocols. By integrating PrEP access through emergency departments, family medicine, and clinical settings offering reproductive health care, we can increase the continuity of care for PrEP and enhance access and the likelihood of PrEP uptake, persistence, and adherence. Future research efforts to integrate PrEP into existing healthcare settings must also motivate healthcare providers to engage PrEP-eligible populations in conversations about PrEP and facilitate linkage to this important biomedical intervention.

## Conclusion

Although iPrEP appeared to have an inverse effect on readiness and willingness to take PrEP when compared to usual care, further utility of this approach is not futile. In future iterations, it is imperative that revisions to the iPrEP tool carefully considers the level of transparency on side effects and daily pill burden needed to motivate PrEP uptake among cisgender Black women. Approaching the content in a similar way that clinicians approach transparency when sharing information about clinical procedures (i.e. surgeries) to patients should be mobilized. Interventionists should be careful to assess the type of knowledge content that aligns with motivation among cisgender women through future qualitative approaches. Researchers designing interventions for cisgender women aiming to increase PrEP access must also pay close attention to the intervention approach used to deliver PrEP related content. As uptake of PrEP is already low among populations who could benefit from it most, specifically cisgender Black women, researchers and interventionists must be very careful to prevent de-motivating this population from accessing PrEP, as this could stave off progress towards our nation’s Ending the HIV Epidemic plan.

Educational material regarding PrEP as a bi-monthly injection should be integrated into revised versions of iPrEP as an intervention tool. Future revisions to the iPrEP intervention will encompass the health benefits of HIV prevention and the ability to lower risk of disease transmission, with an emphasis on HIV through PrEP uptake. Revising and refining approaches that can sexually liberate cisgender Black women, while equipping this population with effective prevention tools, is an avenue with the capacity to rid our health communication of stigma and further marginalization when engaging the healthcare system in prevention services.

## Data Availability

The dataset used and/or analyzed during the current study are available from the corresponding author on reasonable request.

## Abbreviations

PrEP: Pre-exposure prophylaxis
ED: Emergency department
iPrEP: Increasing willingness for PrEP intervention
RCT: Randomized controlled trial
LBJ: Lyndon B. Hospital
MHH: Memorial Hermann Health System
EMR: Electronic medical record
CDC: Centers for Disease Control and Prevention
ID: Identification
HPTN: HIV Prevention Trials Network
MSM: Men who have sex with men
RAB: Risk assessment battery
TLFB: Timeline follow-back
GLM: Generalized linear modeling
PP: Posterior probability
Crl: Credible interval
